# Mouse strain differences in response to oral immunotherapy for peanut allergy

**DOI:** 10.1002/iid3.242

**Published:** 2019-03-05

**Authors:** Laura Wagenaar, Marianne W.H.C. Bol‐Schoenmakers, Giulio Giustarini, Johan Garssen, Joost J. Smit, Raymond H.H. Pieters

**Affiliations:** ^1^ Faculty of Veterinary Medicine Department of Immunotoxicology Institute for Risk Assessment Sciences Utrecht University Utrecht The Netherlands; ^2^ Faculty of Science, Department of Pharmacology, Utrecht Institute for Pharmaceutical Sciences Utrecht University Utrecht The Netherlands; ^3^ Department of Immunology Nutricia Research Utrecht The Netherlands

**Keywords:** BALB/c, C3H/HeOuJ, immunotherapy, mouse model, peanut allergy

## Abstract

**Background:**

Promising therapies for food allergy are emerging, mostly based on animal experimentation. However, different mouse strains are used, which may make it hard to compare experiments. The current study investigated whether the immunological differences between C3H/HeOuJ (C3H) and BALB/c mice lead to differences in efficacy of peanut‐specific immunotherapy.

**Methods:**

After sensitization using peanut extract (PE), C3H and BALB/c mice received oral immunotherapy (OIT) by intragastric dosing for three weeks. Hereafter, mice were exposed to PE via the intradermal, intragastric and intraperitoneal route, to determine allergic outcomes. Furthermore, PE‐specific antibody and cytokine production were determined and the number of various immune cells at different time points during the study were measured.

**Results:**

OIT protected C3H mice against anaphylaxis, whereas no anaphylaxis was seen in BALB/c mice. In contrast, OIT induced an increase in MMCP‐1 levels in BALB/c mice but not in C3H mice. No effect of OIT on the acute allergic skin response was observed in either strain. Specific antibody responses showed similar patterns in both strains for IgA and IgG1. IgE levels were a tenfold higher in BALB/c mice and after the intragastric challenge (day 70) OIT‐treated BALB/c mice showed induced IgE levels. Moreover, in C3H mice IgG2a levels were higher and increased in response to OIT and challenges. After the final challenge, but not at other timepoints MLN‐derived lymphocytes from OIT‐treated BALB/c mice produced less IL‐13 and IL‐5 compared to control‐treated mice, whereas no differences were seen in case of C3H mice.

**Conclusions:**

Taken together, these results show that the C3H strain is more suitable to study clinical outcomes of OIT, whereas the BALB/c strain is more optimal to study T cell responses.

**Abbreviations**
AIT: antigen‐specific immunotherapy
C3H: C3H/HeOuJ
CT: cholera toxin
i.d.: intradermal
i.g.: intragastric
i.p.: intraperitoneal
Ig: immunoglobulin
IL: interleukin
IFN: interferon
MMCP‐1: murine mast cell protease‐1
OIT: oral immunotherapy
PE: peanut extract

## 1 INTRODUCTION

Peanut allergy is a serious and sometimes life‐threatening condition. The prevalence of food allergies has increased to 6% in children and 3% in adults. In particular, the incidence of peanut allergy doubled in American children less than 5 years of age in the past 5 years.^1^, ^2^ Although food allergies such as milk allergy are generally outgrown, peanut allergy is persistent to adulthood and represents a major cause of food‐induced anaphylaxis.^3^, ^4^

Allergen‐specific immunotherapy (AIT) has been used for over a 100 years and forms a long‐term treatment for allergies.^5^ Early studies of subcutaneous immunotherapy (SCIT) in patients with peanut allergy were discontinued because of a high rate of anaphylactic reactions.^6^, ^7^ Oral immunotherapy (OIT), on the other hand, has demonstrated to potentially desensitize patients to specific food allergens.^8^ The success rates of OIT vary from 36% to 100% for desensitization and 28% to 75% for tolerance,^9^ illustrating the wide variation in responses to OIT among patients.^10^ Whether this variation is caused by genetic differences or different mechanisms behind OIT play a role is currently unknown.

Animal models have highly contributed to the insight in mechanisms of food sensitization, and more recently, also in the mechanisms involved in AIT and OIT. In most models, C3H/HeOuJ or C3H/HeJ (C3H) mice or BALB/c mice are used, but differences in allergic manifestations exist between both strains.^11^ For example, C3H mice show clinical anaphylactic responses upon allergen exposure whereas BALB/c mice do not.^11^, ^12^, ^13^, ^14^, ^15^ However, to our knowledge, the response to AIT has never been compared in both strains in the same study. Investigating this responsiveness to AIT in different strains of mice will not only elucidate differences in the mechanism of immune responses in AIT, but also improve the selection of the appropriate mouse strains to test interventions.

Here, we compared the efficacy of OIT in C3H and BALB/c mice using a model as described before.^16^ Marked differences with regard to the extent of oral sensitization to peanut were observed between both strains. Interestingly, after OIT anaphylactic and T cell responses differed significantly between both strains. Ultimately this finding and future research into strain differences may be of relevance to the human situation where inter‐individual differences might contribute to the sometimes‐limited success of therapeutic approaches for food allergy.

## 2 MATERIALS AND METHODS

### 2.1 Mice

All animal procedures were approved by an independent ethics committee for animal experimentation (Ethical Committee of Animal Research of Utrecht University, Utrecht, The Netherlands, registered by DEC2014.III.12.120), and complied with the principles of good laboratory animal care following the European Directive for the protection of animals used for scientific purposes (2010/63/EU). Specific‐pathogen free 6‐week old female C3H/HeOuJ and BALB/c mice (total *n* = 72, *n* = 6 per group) were purchased from Charles River Laboratories (Erkrath, Germany). The animals were housed at the animal facility of Utrecht University on a 12 h light/dark cycle, in filter‐topped macrolon cages (*n* = 6 per cage) on woodchip bedding. Food and water were provided ad libitum.

### 2.2 Reagents

Peanut protein extract (PE) was prepared from raw peanuts (provided by Intersnack Nederland BV, The Netherlands) as described previously.^17^ The extract contained 30 mg/mL protein as determined by Bradford analysis with Bovine Serum Albumin (BSA) as a standard. Cholera toxin (CT) was purchased from List Biological Laboratories (Inc, Campbell, CA).

### 2.3 Experimental set‐up: oral sensitization, immunotherapy, and challenges of mice

**Figure 1:**
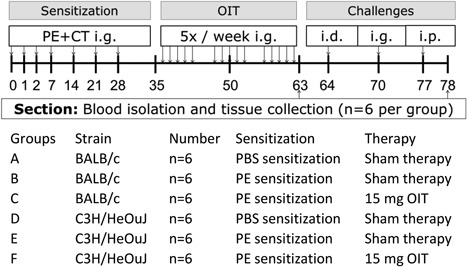
Experimental set‐up of the peanut allergy model. C3H/HeOuJ and BALB/c Mice were sensitized i.g. to PE or PBS in combination with CT. From day 42, the mice were treated orally for five times/week with allergen or PBS alone, for three consecutive weeks. On day 65, all mice were challenged i.d. to determine the acute allergic skin response. On day 70 an i.g. challenge was performed to measure mucosal mast cell degranulation. After the i.p. challenge on day 77 anaphylactic shock symptom scores and body temperature levels were measured. Mice were divided in two dissection cohorts (day 63 and day 78), every cohort contained all study groups. On day 63 and 78 the cohort of that day was killed with cervical dislocation and blood and organs were collected. PE, peanut extract; d, day; CT, cholera toxin; OIT, oral immunotherapy; i.d., intradermal; i.g., intragastric; i.p., intraperitoneal

The experimental design is depicted in Figure 1. After randomization and acclimatization (1 week), mice were sensitized intragastrically (i.g.) to PE (6 mg in 200 μL PBS) using CT (15 µg/mouse) as an adjuvant, according to the method described by van Wijk et al (day 0, 1, 2, 7, 14, 21, and 28).^18^ Sham‐sensitized mice were treated with CT in PBS alone. Starting on day 42, the mice were dosed i.g. with the oral immunotherapy treatment (OIT, 15 mg PE in 500 μL PBS) five times/week, for 3 weeks (day 42–60). Sham‐sensitized and allergen‐sensitized control mice were treated i.g. with PBS alone.

To determine ear swelling as a measure for the acute allergic skin response (day 64), all mice were anesthetized by inhalation of isoflurane to determine ear thickness in duplicate prior to, and 1 h after an intradermal injection in both ear pinnae with PE (1 μg PE in 20 µL PBS). Ear thickness was measured with a digital micrometer (Mitutoyo, Veenendaal, The Netherlands). Basal ear thickness (µm), was subtracted from the ear thickness 1 h post‐challenge.

On day 70, 30 min after a i.g. challenge (15 mg PE in 500 μL PBS) blood samples were collected, to measure mast cell degranulation.

Anaphylactic shock symptom scores and body temperature levels were measured after an intraperitoneal (i.p.) challenge on day 77 (100 μg PE in 200 μL PBS). Every 10 min after the i.p. challenge body temperature was measured using a rectal thermometer. After 40 min symptoms of anaphylaxis were scored, according to the method described by Smit et al.^11^

After OIT (day 63), and at the end of the experiment (day 78), mice were killed by cervical dislocation and blood and organs were collected.

### 2.4 Levels of Mouse Mast Cell Protease‐1 (MMCP‐1) and allergen‐specific IgE, IgA, IgG1 and IgG2a in serum

Blood samples were collected via cheek puncture at five time points during the animal experiment (day 35, 50, 64, 70, 78). After centrifugation (10 000 rpm for 7 min) sera were stored at −20°C until antibody analysis.

PE‐specific IgA, IgE, IgG1, and IgG2a levels in serum were measured by ELISA as previously described.^11^ For IgE and IgA, 96‐wells high‐binding plates (Costar 3590, Corning Incorporated, Corning, NY) were coated with 1 μg/mL rat anti‐mouse IgE or IgA (BD Biosciences, Alphen aan de Rijn, The Netherlands). For IgG1 and IgG2a plates were coated with 10 μg/mL PE in PBS overnight at 4°C. After 1 h of blocking (room temperature) with 0.5% BSA‐ELISA buffer (50 mM TRIS, 137 mM NaCl, 2 mM EDTA and 0.05% Tween20), diluted serum samples were incubated for 2 h at room temperature. AP‐coupled anti‐IgG1 and IgG2a were added (1 h RT) for detection, followed by 1 mg/mL p‐nitrofenylphosphat in diethanolamine buffer for the color reaction, which was stopped with a 10% EDTA solution. Absorbance was measured at 405 nm using an Asys expert 96 plate reader (Biochrom, Cambourne, UK). For detection of PE‐specific IgE and IgA, PE‐DIG conjugate solution (1 h RT) and peroxidase‐conjugated anti‐DIG fragments (1h at RT in the dark) were used. After incubation, a tetramethylbenzidine substrate solution was added, the color reaction was stopped with 2M H_2_SO_4_, hereafter absorbance was measured at 450 nm. Concentrations of the antibodies were calculated in arbitrary units (AU) using a standard curve of pooled sera from PE‐sensitized mice.

MMCP‐1 was determined by using a sandwich ELISA kit (eBioscience Mouse MCPT‐1 ELISA Ready‐SET‐Go Kit, Breda, The Netherlands) according to the manufacturer's instructions. Levels of MMCP‐1 were measured in serum samples collected 30 min after the i.g. challenge.

### 2.5 Cell analysis using flow cytometry

Lymphocytes from the mesenteric lymph nodes (MLN) and spleen were obtained by squeezing the organs through a 70‐µm strainer. Red blood cells from spleen suspension were lysed, using an erylysis buffer (containing NH4Cl, KHCO3, and Na2EDTA) for 1 min, and washed once in PBS.

5–10 × 10^5^ cells per well were collected in fluorescence activated cell sorting (FACS) buffer (PBS containing 0.25% BSA, 0.05% NaN3, 0.5 mM EDTA) and plated for flow cytometry analysis. Fc‐receptor was blocked using anti‐CD16/CD32 (clone 2.4G2). Hereafter, cells were incubated with fluorescent‐labeled antibodies. Cells were fixed using 0.4% paraformaldehyde or, for regulatory T cell staining, cells were permeabilized and fixed using the buffer set purchased from eBioscience according to the manufacturer's protocol.

The antibodies used were: anti‐CD4‐FITC (1:200, clone RM4‐5), anti‐FoxP3‐APC (1:40, clone FJK‐16s), anti‐CD25‐PE (1:200, clone PC61.5). Acquisition of the samples was performed on the BD Accuri™ C6 flow cytometer, analysis with BD sampler software (BD Biosciences). Cut‐off gates for positivity were established using the fluorescence‐minus‐one (FMO) technique.

### 2.6 Cytokine release after ex vivo stimulation with PE and αCD‐3/CD‐28

Cells derived from the MLN were cultured in U‐bottom culture plates (Greiner, Frickenhausen, Germany) at 8 × 10^5^ cells per well in culture medium (RPMI 1640 medium (Lonza, Verviers, Belgium) with 10% FCS, penicillin (100 U/mL)/streptomycin (100 µg/mL, Sigma)). Cells were stimulated with culture medium as a negative control, a polyclonal stimulation with anti‐CD3/CD28 (1 µg/mL, clone 145‐2C11 and clone 37.51, eBioscience) or antigen‐specific stimulation with PE (100 µg/mL). To assess production of Interleukin (IL)‐5, IL‐10, IL‐13, and Interferon‐γ (IFN‐γ) by T cells, cells were incubated for 48 h (anti‐CD3/CD28) or 96 h (PE). Culture supernatants were collected and stored at −20°C until further analysis with the Ready‐SET‐Go!® ELISA (eBioscience) according to the manufacturer's instructions.

### 2.7 Basophil activation assay

Blood was taken from the mice on day 56, stimulated, and analyzed for basophil activation as previously described by Torrerro et al.^19^ In summary, whole blood was diluted 1:1 using RPMI in heparinized tubes. After incubation with anti‐mouse IgE at 0.125 µg/mL (R35‐72, BD Biosciences) or PE at 20 µg/mL for 90 min at 37°C in 5% CO_2_, activation was stopped with PBS containing EDTA in 12 × 75 mm test tubes. The Whole Blood Lysing Reagents was used to lyse the red blood cells and fix the samples according to protocol (Beckman Coulter, Fullerton, CA). To block the Fc‐receptor, cells were incubated with anti‐CD16/CD32 (clone 2.4G2) for 20 min. Cells were stained for 30 min at 4°C with the following fluorescent‐labeled antibodies: anti‐IgE‐FITC (1:100, clone 23G3), anti‐CD49b‐APC (1:200, clone CX5), anti‐CD4‐PE (1:200, clone RM4‐5), anti‐CD200R‐Percpefluor 710 (1:200, clone OX110), and anti‐CD19‐PE (1:200, clone 6D5) from eBioscience. Acquisition of the samples was performed on the BD Accuri™ C6 flow cytometer, analysis with BD sampler software (BD Biosciences). Fluoresence minus one technique was used to set the gates.

### 2.8 Statistics

For all statistical analyses, GraphPad Prism 6.0c software for Macintosh (GraphPad Software, San Diego, CA) was used. Data are depicted as mean ± SEM. Cytokine levels were log‐transformed before statistically analyzed. The acute allergic skin response, serum MMCP‐1 levels and immunoglobulin levels on each day were statistically analyzed using one‐way ANOVA and Dunnet's post‐hoc test for multiple comparisons to compare selected groups. Body temperature levels were statistically analyzed using repeated measures two‐way ANOVA and Bonferroni's post‐hoc test. Flow cytometry data and cytokine levels were statistically analyzed using two‐way ANOVA and Dunnett's post‐hoc test for multiple comparisons. Anaphylaxis symptom scores were analyzed using a Kruskal‐Wallis test for nonparametric data with Dunn's post hoc test. All data are presented as mean ± SEM of six animals per group and results were considered statistically significant when *P* < 0.05.

## 3 RESULTS

### 3.1 Systemic anaphylaxis after OIT and challenges is mouse strain‐dependent

**Figure 2:**
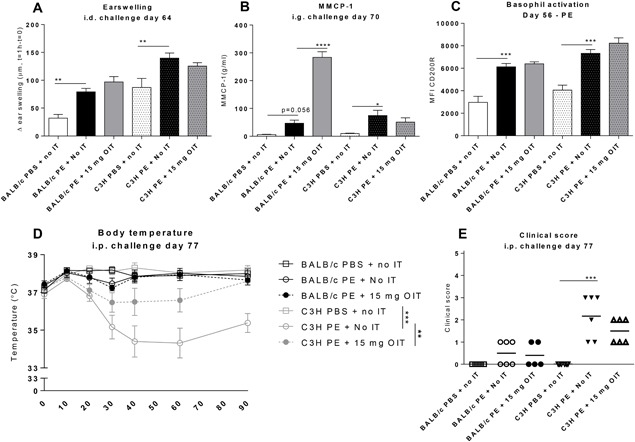
Allergic manifestations in PE‐sensitized mice after receiving OIT. A, Acute allergic skin response measured as Δ ear swelling 1 h after i.d. challenge. B, Concentrations of MMCP‐1 in serum collected 30 min after i.g. challenge. C, On day 56 peripheral blood basophil activation was measured after whole blood stimulation with PE using flow cytometry. Cells were gated based on FSC‐SSC properties and the Fluorescence‐minus‐one (FMO) technique. Results are indicated as mean MFI CD200R+ of CD19/CD4−, IgE + CD49b+ cells. D, Change in body temperature after i.p. challenge. E, Anaphylactic shock symptom scores determined 40 min i.p. challenge. Data are represented as mean ± SEM *n* = 6 mice/group. Statistical analysis was performed using one‐way ANOVA and Dunnet's post hoc test for multiple comparisons (earswelling, MMCP‐1and basophil activation), a Kruskal‐Wallis test with Dunn's post hoc test (clinical score) or a two‐way ANOVA for repeated measures (body temperature). *P < 0.05; **P < 0.01; ***P < 0.005 compared to indicated group. OIT, oral immunotherapy; PE, peanut extract; CT, cholera toxin; MMCP‐1, mucosal mast cel protease‐1; MFI, mean fluorescence intensity; IT, immunotherapy

The acute allergic skin response, as characterized by ear swelling upon intradermal challenge with PE, was similar in both strains (Figure 2A), and this response was not changed after OIT.

To determine the effect of OIT on the gastrointestinal mast cell response, MMCP‐1 levels were measured in serum collected 30 min after an intragastric challenge (Figure 2B). In the C3H mice, PE sensitization significantly induced MMCP‐1 compared to sham‐sensitized controls. Remarkably, OIT increased MMCP‐1 in BALB/c mice but did not change MMCP‐1 levels in C3H mice. PE‐specific activation of basophils measured upon isolation on day 56, was significantly increased in C3H mice, but not in BALB/c mice in comparison to sham‐sensitization (Figure 2C). OIT did not alter PE‐specific basophil activation. αIgE‐mediated stimulation of these basophils was not significantly different between strains or treatments (data not shown). As we have shown before,^11^ a clear anaphylactic response, characterized by a drop in body temperature and by anaphylactic shock symptoms was observed in PE‐sensitized C3H mice, but absent in BALB/c mice (Figures 2D and 2E). Importantly, OIT was able to protect C3H mice from this anaphylactic response.

### 3.2 Increased levels of allergen‐specific IgE in BALB/c mice after OIT while in C3H mice IgG2a levels were higher and increased in response to OIT and challenges

**Figure 3:**
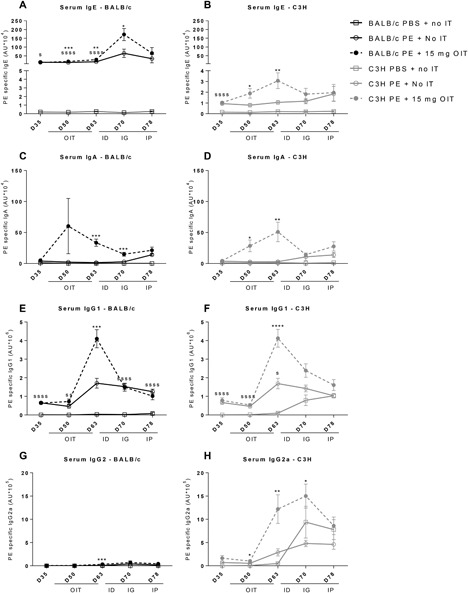
Allergen specific IgE, IgA, IgG1, and IgG2a levels in serum. PE sensitized mice received OIT for 3 weeks. Blood was taken on d35, d50, d63, d70, and d78. A‐D, Allergen‐specific IgE, IgA, IgG1, and IgG2a were measured in serum by ELISA. Data are represented as mean ± SEM *n* = 6 mice/group. Statistical analysis was performed using a one‐way ANOVA on each time‐point and Dunnet's post hoc test for multiple comparisons. *P < 0.05; **P < 0.01; ***P < 0.005; ****P < 0.0005 compared to PE sensitized control group. SS *P* < 0.01; SSSS *P* < 0.001; compared to the PBS sensitized control group. OIT, oral immunotherapy; PE, peanut extract; id, intradermal challenge; ig, intragastric challenge; ip, intraperitoneal challenge

PE‐sensitization increased IgE and IgG1 in both strains (day 35 Figures 3A, 3B, 3E, and 3F). OIT increased IgE (day 50 and 63), IgA (day 50 and 63), IgG1 (day 63) and IgG2a (day 50, 63, and 70) in C3H mice (Figure 3B, 3D, 3F, and 3H). Similarly, OIT in BALB/c mice increased PE‐specific IgE (day 50, 63, and 70), IgA (day 63 and 70), IgG1 (day 63), and IgG2a levels (day 63, Figures 3A, 3C, 3E, and 3G). However, IgE levels were over a tenfold higher in BALB/c mice than in C3H, whereas IgG2a levels were higher in the C3H strain (Figure 3). Moreover, IgE levels o increased to a maximum on day 70 (after the i.g. challenge) in OIT‐treated BALB/c mice, whereas in C3H mice levels were similar between OIT‐treated and non‐OIT treated sensitized mice (day 70, Figures 3A and 3B).

### 3.3 Decreased percentages of Tregs in both strains after OIT

**Figure 4:**
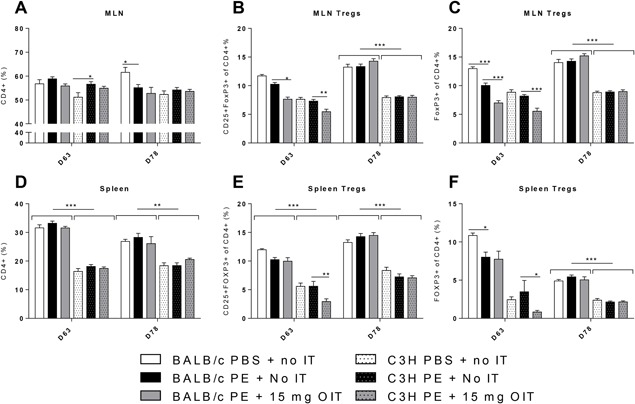
Flow cytometric analysis of T cell populations in the spleen and MLN. PE sensitized mice received OIT for three weeks. On day 63 and day 78 MLN and Spleen lymphocytes were isolated. Cells were gated based on FSC–SSC properties and the Fluorescence‐minus‐one (FMO) technique. A and E, Percentage of CD4+ cells in MLN and Spleen. B and E, Percentage of Tregs (CD25 + FoxP3+ of CD4 +) in MLN and spleen. C and F, Percentage of activated Tregs (FoxP3+ of CD4 +) in MLN and spleen. All data are represented as mean ± SEM *n* = 6 mice/group. Statistical analysis was performed using two‐way ANOVA and Dunnett's post hoc test for multiple comparisons. *P < 0.05; **P < 0.01; ***P < 0.005; compared to indicated group

Total CD4+ cell percentages were not different between strains, but the percentages of the Treg subsets (CD4 + CD25 + FoxP3 +) and (CD4 + FoxP3 +) in MLN were higher in the BALB/c mice compared to C3H mice irrespective of treatment (Figures 4B and 4C). In the spleen, percentages of CD4+ and Treg cells were higher in BALB/c mice than in C3H mice (Figures 4D, 4E, and 4F).

OIT lowered the percentages of Treg cells in the MLN in both strains and in the spleen of C3H mice (day 63, Figures 4B, 4C, 4E, and 4F). This decrease was apparent immediately after treatment (day 63), whereas on day 78 the amounts of these regulatory cells was not different from controls.

### 3.4 Decreased Th2 cytokine levels after OIT in BALB/c mice

**Figure 5:**
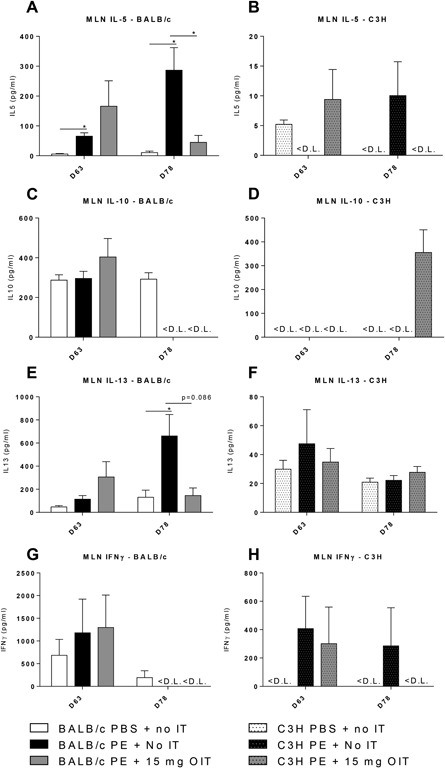
Cytokine concentrations after ex vivo stimulation of MLN derived lymphocytes with PE, determined by ELISA. PE sensitized mice received OIT for 3 weeks. MLN lymphocytes from different days were cultured for 96 h in the presence of PE or medium (medium data not shown). A and B, IL‐5 concentration. C and D, IL‐10 concentration. E and F, IL‐13 concentration. G‐H IFNγ concentration in MLN. Data are represented as mean ± SEM *n* = 6 mice/group. <L.D., below the limit of detection. Statistical analysis was performed using one‐way ANOVA and Dunnett's post hoc test for multiple comparisons. *P < 0.05 compared to indicated group

Cytokine production was measured in the culture supernatant of PE‐stimulated lymphocytes obtained from the MLN on day 63 and 78 (Figure 5). The levels and responses of cytokines between the two mouse strains were clearly different, with higher levels of IL‐5, IL‐10, IL‐13, and IFN‐ γ produced by lymphocytes of BALB/c compared to lymphocytes of C3H. Cytokine levels in cultures of MLN obtained from C3H mice were mostly below or around the limit of detection (Figures 5B, 5D, 5F, 5H). In contrast, ex vivo PE stimulation of lymphocytes of BALB/c mice obtained on day 78 resulted in increased IL‐5 and IL‐13 release in PE‐ sensitized mice compared to sham‐sensitized mice (Figures 5A and 5E). However, lymphocytes obtained from OIT‐treated mice on day 78 showed lower levels of IL‐5 and IL‐13, when compared to PE‐sensitized control mice (Figures 5A and 5E). A difference in IL‐5 and IL‐13 production between day 63 and 78 can be seen (Figures 5A and 5E); on day 63 OIT induces an increase in IL‐5 and IL‐13 (although not significant), while after the PE challenge regime (day 78) IL‐5 and IL‐13 are increased in PE‐sensitized control mice and this increase is not observed in OIT treated mice (Figures 5A and 5E). Production of IL‐10 and IFN‐γ were not different between BALB/c treatment groups (Figures 5C and 5G).

## 4 DISCUSSION

Peanut allergy is an important public health problem because it affects children and adults, can be severe and even life‐threatening, and may be increasing in prevalence in westernized countries.^20^ Therefore, rapid advances in the knowledge on developing effective therapeutic strategies as well as on mechanisms are needed. Intervention trials in humans can be hazardous because of the risk of adverse reactions, which make well‐characterized mouse models that reflect human food allergy highly desirable. Selecting the most appropriate model to study food allergy interventions is important since it allows better comparison of various therapeutic strategies and different mechanisms involved. In addition, it is important that these models show clinical effects comparable to that of patients, to enable prediction of efficacy of the intervention.

Previously, our group has demonstrated that the occurrence of systemic anaphylaxis after peanut sensitization is strain‐dependent and most severe in C3H, less pronounced in C57BL/6 and very low or minimal in BALB/c mice.^11^ Also Morafo et al has shown that BALB/c mice did not demonstrate an allergic response upon challenge after peanut or cow's milk allergy sensitization.^21^ Similar results were shown after i.p. sensitization using alum as an adjuvant, however, this method did result in a small systemic anaphylaxis reaction in BALB/c.^22^ When both strains were sensitized to ovalbumin, BALB/c mice exhibited similar anaphylactic symptoms as C3H mice, but no effects were seen on body temperature.^23^ Also, in the current study strain differences regarding sensitization were clearly visible: no anaphylaxis (ie, no clinical score, no temperature drop) occurred in BALB/c mice, whereas in C3H mice it did. Moreover, IgE levels after sensitization were higher in BALB/c mice compared to C3H mice.

These differences were also reflected in strain‐specific responses to OIT. C3H mice showed a decrease in anaphylactic response to OIT which is in accordance with our previous data,^16^ whereas BALB/c mice showed clear effects of OIT on mostly Th2‐linked antibody and cytokine responses. For BALB/c mice the duration of treatment may be of importance since others have shown that BALB/c mice sensitized to cow's milk showed anaphylactic symptoms following an oral exposure, but these symptoms were lowered by long‐term OIT administration.^24^ Moreover, choosing another clinical readout could prove clinical efficacy in BALB/c mice, Burggraf et al showed that after intraperitoneal sensitization with OVA, OIT improved inflammation in the small intestines and gastrointestinal symptoms in BALB/c mice.^25^

Antibody responses were also different between the two strains. After sensitization, OIT and subsequent allergen challenges levels of IgE and IgG2a clearly differed between the strains. I.p. sensitization resulted in higher IgG1 and IgG2a levels after sensitization in C3H mice compared to BALB/c mice,^22^ whereas we measured comparable levels after sensitization in both strains. Interestingly, OIT treatment resulted in a significantly higher IgG2a responses in C3H mice compared to BALB/c mice. Whether this implicates a (mechanistic) role for IgG2a in the protection to allergic manifestations by OIT as observed in C3H mice remains to be investigated. Conversely, epicutaneous immunotherapy for different allergens in BALB/c mice increased IgG2a levels but not IgG1 levels and the response of sensitized animals to allergen‐induced bronchial hyper responsiveness was reduced in these mice.^26^

It has been shown that IgG antibodies can suppress IgE‐mediated hypersensitivity,^27^ by capturing of allergens before binding to IgE present on mast cells and basophils hereby preventing degranulation.^28^ In humans, OIT is known to cause initial increases of antigen‐specific IgE, followed by a decline after prolonged treatment.^29^ Similar to the human situation, we here showed that IgE levels in C3H mice initially increase after OIT but decrease again after allergen challenges (apparent on day 70). In contrast, the increased IgE levels seen in BALB/c mice, could explain the higher mucosal mast cell degranulation evidenced by higher MMCP‐1 levels after i.g. challenge. We hypothesize that the differences in antibody levels might be caused by differences in number or activation of B cells or mast cells between the two strains. Female BALB/cByJ mice showed a tenfold higher relative percentage of B cells compared to female C3H/HeJ mice.^30^ Moreover, when comparing BALB/c and C57BL/6 mice, it was demonstrated that BALB/c mice showed increased number of plasma cells and increased B‐cell activation, after immunization with T‐dependent antigens which may emphasize the importance of examining B‐cell behavior in the context of the strain used.^31^

After PE challenges, in BALB/c mice, peanut recall responses in MLN cultures showed that OIT decreased Th2 cytokine production compared to MLN cultures from PE‐sensitized control mice. This indicates that, after allergen challenges, OIT in BALB/c mice is skewing the immune response away from Th2 type responses profile. In accordance, clinical protection after OIT in peanut allergic patients in a randomized controlled trial was accompanied by a reduction in IL‐5 and IL‐13.^32^ Skewing of the immune response from a Th2 profile toward a more regulatory profile is associated with a modified cytokine milieu.^33^ However, on day 63, we did not observe a decrease of IL‐5 and IL‐13 after OIT treatment as seen on day 78, indicating that OIT rather prevented the increase in Th2 cytokine responses seen after PE challenges. BALB/c mice produce more of all cytokine types compared to C3H mice, indicating that genetic differences play a role. This finding may be relevant for translation of effects to the human situation since inter‐individual differences may be the cause of sometimes inconclusive diagnosis of and limited success of therapeutic approaches for food allergy.

In the spleen, CD4+ T cell percentages are higher in all BALB/c groups compared to the C3H groups and this difference is also seen in Treg cell percentages. Nevertheless, in both strains OIT caused a decrease of Treg percentages in both MLN and spleen after therapy. In contrast, Dioszeghy et al showed in BALB/c mice that 8 weeks OIT, but also EPIT and sublingual immunotherapy increased Treg cell percentages in the spleen, accompanied by similar results on IgG2a levels and cytokine production.^15^ This suggests that different OIT dosing schedules could induce different effects on T‐reg cell subsets in different mouse strains. We speculate that the observed drop in Treg number in the MLN on d63 in both strains can be explained by an efflux of these cells to an effector site, for example, the intestinal lamina propria. This speculation is based on our previous results obtained in a cow's milk allergy model that show an increase in the percentage of Foxp3+ Tregs in the lamina propria of OIT‐treated mice compared to sensitized control mice.

In conclusion, this study shows that allergic responses after OIT are clearly different between peanut‐sensitized C3H and BALB/c mice. A particularly interesting finding was the more pronounced T cell response in BALB/c mice compared to C3H mice, with a reduction in Th2 response due to OIT in the MLN of BALB/c mice. However, the absence of clinical manifestations in BALB/c mice after oral peanut challenge indicate that the C3H strain is more appropriate to study improvement of clinical responses by OIT. Collectively, our study suggest that the C3H/HeOuJ strain is more suitable to study clinical outcomes of OIT, whereas BALB/c mice will be the more appropriate strain if the cellular response is to be assessed.

## ETHICS APPROVAL

All experimental procedures were approved by the Ethical Committee of Animal Research of Utrecht University and complied with the principles of good laboratory animal care following the European Directive for the protection of animals used for scientific purposes (registered by AVD108002015212).

## FUNDING SOURCES

This research was financially supported by the STW ‘Open Technology Program’ grant and embedded in the NUTRALL consortium project entitled: “Nutrition‐based approach to support antigen‐specific immunotherapy for food allergies” (grant number 12652).

## AVAILABILITY OF DATA AND MATERIALS

The datasets used and/or analyzed during the current studies are available from the corresponding author on reasonable request.

## CONFLICT OF INTEREST

None of the authors have a competing financial interest in relation to the presented work; JG is partly employed by Nutricia Research, Utrecht, The Netherlands.
